# The relationship between gut microbiota, short-chain fatty acids and type 2 diabetes mellitus: the possible role of dietary fibre

**DOI:** 10.1007/s00592-021-01727-5

**Published:** 2021-05-10

**Authors:** Dominic Salamone, Angela Albarosa Rivellese, Claudia Vetrani

**Affiliations:** 1 Department of Clinical Medicine and Surgery, University of Naples “Federico II”, 5 Sergio Pansini, 80131 Naples, Italy; 2 Task Force On Microbiome Studies, University of Naples “Federico II”, Naples, Italy

**Keywords:** Gut microbiota, Short-chain fatty acids, Type 2 Diabetes, Insulin resistance, Glucose tolerance, Dietary fibre

## Abstract

Gut microbiota and its metabolites have been shown to influence multiple physiological mechanisms related to human health. Among microbial metabolites, short-chain fatty acids (SCFA) are modulators of different metabolic pathways. On the other hand, several studies suggested that diet might influence gut microbiota composition and activity thus modulating the risk of metabolic disease, i.e. obesity, insulin resistance and type 2 diabetes. Among dietary component, dietary fibre may play a pivotal role by virtue of its prebiotic effect on fibre-fermenting bacteria, that may increase SCFA production. The aim of this review was to summarize and discuss current knowledge on the impact of dietary fibre as modulator of the relationship between glucose metabolism and microbiota composition in humans. More specifically, we analysed evidence from observational studies and randomized nutritional intervention investigating the relationship between gut microbiota, short-chain fatty acids and glucose metabolism. The possible mechanisms behind this association were also discussed.

## Introduction

The gut microbiota is a complex and dynamic ecosystem that interacts with the host while maintaining a mutualistic relationship with it. Indeed, it may influence multiple physiological mechanisms related to human health, i.e. synthesis of micronutrients, defence against pathogens, regulation of glucose and lipid metabolism, and immune function [1].

Therefore, it has been suggested that the modulation of gut microbiota could be a reliable tool to prevent metabolic and inflammatory diseases. In particular, animal studies support a causal role between the composition of the gut microbiota and development of obesity, insulin resistance and type 2 diabetes (T2D). In addition, observational studies have confirmed the presence of altered gut microbiota composition, named “*dysbiosis*”, in prediabetic or T2D patients compared to healthy subjects [2, 3]. However, no specific microbial communities related to the onset of these diseases have been identified so far.

Nevertheless, more evidence is available on the microbial activities linked to the beneficial effect of the gut microbiota against T2D [4]. The main mechanisms can be summarized as follows: (1) maintenance of the integrity of intestinal barrier; (2) reduction in bacteria translocation and, consequently, systemic inflammation (endotoxemia); (3) production of short-chain fatty acids (SCFA) (acetate, propionate and butyrate) which can influence metabolic pathways [5].

The first two effects (maintenance of intestinal barrier and reduction in endotoxemia) seem to be more closely linked to the onset of diseases. Conversely, the production of SCFA could represent a tool to both prevent and modulate T2D [6, 7].

Against this background, several researches have been carried out to identify potential strategies to induce specific changes in the gut microbiota composition towards microbial species with high fermentative activity.

Gut microbiota composition is influenced by internal and external factors. Recently, it was shown that genetics plays a marginal role in the definition of microbiota composition [8]. As for external factors, faecal transplantations and antibiotics have dramatic but temporary effects on the host microbiota [9]. Conversely, dietary changes could be more effective to induce lasting changes in the composition of gut microbiota. Among dietary components, dietary fibres play a pivotal role. In fact, it is well known that fibres are the main substrate for bacterial metabolism. Furthermore, recent findings demonstrated that habitual fibre intake can endorse a “virtuous cycle”, consisting in the overgrowth of fibre-fermenting microbial groups while inhibition of other species [10].

Within this context, the prebiotic effect of dietary fibres might be a feasible strategy to prevent T2D, through the modulation of metabolic response. However, to date, there is no conclusive evidence to support this thesis, likely due to the lack of studies focusing primarily on the relationship between the composition of the gut microbiota and metabolic response.

Therefore, in this review, we summarized current evidence from observational and intervention studies performed in humans investigating the relationship between the composition of the gut microbiota, concentration of SCFA and glucose metabolism. Furthermore, the possible mechanisms underlying this association were also discussed.

## Methods

Literature searching for this review was conducted by searching PubMed database for observational studies and randomized controlled clinical trials on humans adults published in the English language, during the last 20 years. The terms “dietary fibre OR fibre OR fibre-rich diet”, “short-chain fatty acids OR butyrate OR acetate OR propionate”, “microbiota OR microbiome OR bacteria”, “type 2 diabetes OR prediabetes OR glucose intolerance OR insulin resistance OR insulin response”, combined with the Boolean operator “AND”, were employed for the research.

**Fig. 1:**
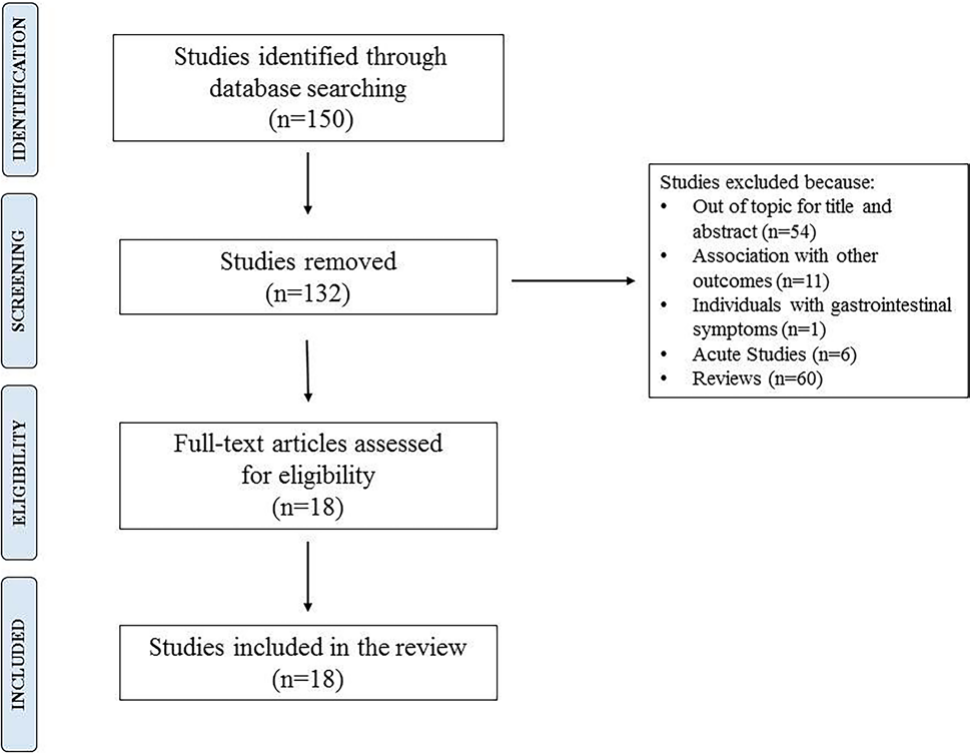
Flow diagram of literature search

This review includes studies published in journals in the highest impact factor quartile in the “Endocrinology and Metabolism” or “Nutrition and Dietetics” areas. We excluded reviews, acute studies and those not specifically related to each issue of interest. Overall, our search retrieved a total of 18 studies suitable for our review, 10 observational studies and 8 randomized controlled trials (Fig. 1).

## Observational studies

Observational studies showed that the composition of the gut microbiota is strictly related to the host metabolic state (Table 1). In particular, two large cohort studies [11, 12] analysed the composition of microbiota into three groups of individuals with different glucose tolerance: normal glucose tolerance, prediabetes and T2D. Greater abundance of butyrate-producing bacteria was observed in individuals with normal glucose tolerance than the other two groups.

**Table 1:** Evidence from observational studies

Reference	Participants	Main results
[11]	44 healthy individuals, BMI 23.4 kg/m^2^ 64 individuals with pre-diabetes, BMI 24.9 kg/m^2^ 13 individuals with T2D, BMI 26.5 kg/m^2^	↑Butyrate-producing bacteria in healthy individuals ↑ *Bacteroides e costridii* in patients with pre-T2D
[12]	206 healthy individuals, BMI 28.2 kg/m^2^ 220 individuals with pre-diabetes, BMI 28.3 kg/m^2^ 58 individuals with T2D, BMI 31.6 kg/m^2^	↓Butyrate-producing Bacteria both in prediabetes and T2D individuals
[13]	53 individuals with T2D, BMI 20–40 kg/m^2^ 49 individuals with pre-diabetes, BMI 20–40 kg/m^2^ 43 healthy individuals, BMI 20–40 kg/m^2^	↑*Roseburia ↑Faecalibacterium prausnitzii* in healthy individuals ↓*Roseburia ↓Faecalibacterium prausnitzi*i in individuals with T2D
[14]	182 healthy individuals, BMI 18-40Kg/m^2^ 183 individuals with T2D, BMI 18-40Kg/m^2^	↑*Roseburia ↑Faecalibacterium praus*nitzii in healthy individuals ↓*Roseburia ↓Faecalibacterium prausnitzii* in individuals with T2D
[15]	952 healthy individuals selected on the basis of genome, metagenomic sequences and SCFA	Butyrate-producing Bacteria play a protective role against T2D
[16]	30 children (1–6 years): 15 African 15 Italian	Plant-based diet (FFQ) ↑P*revotella↑Xylanibacter ↓Firmicutes*
		Animal-based diet (FFQ) ↓*Prevotella ↓Xylanibacter ↑Firmicutes*
[17]	27 healthy individuals, BMI 19–28 kg/m^2^	High MD adherence (FFQ) ↑*Bifidobacteria ↑Bacteroidates* *↓Firmicutes:Bacteroidates* ↑Faecal SCFA ↑Faecal propionate
		High animal protein intake (FFQ) ↓Bac*teroidates* *↑Firmicutes:Bacteroidates*
[18]	31 healthy individuals: 10 healthy individuals with low adherence to MD BMI 21.2–31,2 kg/m^2^ 21 healthy individuals with high adherence to MD BMI 21.6–31 kg/m^2^	High MD adherence (FFQ) ↑Faecal SCFA ↑Faecal propionate ↑Faecal butyrate
[19]	116 healthy individuals, BMI 25–30 kg/m^2^	High MD adherence (FFQ) ↑*Bifidobacteria* ↑Faecal SCFA
[20]	51 vegetarian individuals, BMI 19.4–24.4 kg/m2 51 vegan individuals, BMI 19.1–23.5 kg/m^2^ 51 omnivore individuals, BMI 20.1–24.1 kg/m^2^	Vegetarian Diet, Vegan Diet and Omnivore high MD adherence ↑*Prevotella* ↑Faecal propionate

*BMI* Body mass index; *MD* Mediterranean diet; *FFQ* Food frequency questionnaire, *SCFA* Short-chain fatty acids; *T2D* Type 2 Diabetes.

In line with these results, metagenomic studies showed significant differences in the composition of the gut microbiota of healthy patients compared to diabetic patients in two separate cohorts in China and Europe. In particular, *Roseburia* and *Faecalibacterium prausnitzii* were identified as highly discriminating bacteria between normoglycaemic individuals and those with T2D [13, 14]. In addition, in a cohort of 900 healthy individuals, Senna et al. [15] observed that the abundance of butyrate-producing bacteria is associated with a better insulin response during an oral glucose tolerance test, i.e. a proxy of improved β cell function.

Looking at how dietary habits may influence the composition of the gut microbiota, the study by De Filippo et al. [16] pointed out relevant differences in the composition of gut microbiota in children from Africa and Italy. Specifically, African children had increased *Bacteroides* (mainly *Prevotella* and *Xylanibacter)* and reduced *Firmicutes*, whereas Italian children presented an inverse trend. This finding can be ascribed to different dietary habits in the two cohorts: the former used to a plant-based diet (minor cereals like millet and sorghum, legumes and vegetables) than the latter consuming an animal-based diet rich in fat and protein. This hypothesis is supported by the presence of bacterial strains that hydrolyse fibres, in particular cellulose and xylans, inducing a greater production of faecal SCFA in African children compared to Italian children.

As for other dietary pattern, the relationship between Mediterranean Diet (MD), characterized by a high-fibre intake, and microbial fermentation was explored in different studies. Many authors highlighted that the more adhesion to MD, the greater increase of *Bacteroides* abundance, mainly *Prevotella* [17, 18], or *Bifidobacteria* [19] and *Roseburia* [20]*.* In addition*,* in all these studies individuals more compliant with MD showed increased concentrations of faecal butyrate or faecal propionate [17–20].

## Intervention trials

Randomized nutritional trials focusing on dietary modulation of microbial composition and activity were carried out using high-fibre diets or fibre-rich foods (Table 2).

**Table 2:** Evidence from randomized clinical trials

Reference	Study design	Participants	Duration	Intervention	Observed effects
[21]	Crossover	10 healthy individuals BMI 19–32 kg/m^2^	4 days	Plant-based Diet (fibre: 26 g/1000 kcal) *vs* Western Diet (fibre: 9.3 g/ 1000 kcal)	Plant-based diet ↑*Prevotella* ↑*Roseburia* ↑Faecal butyrate Western diet ↓*Prevotella*↑*Bacteroides*
[22]	Parallel	29 individuals with at least one MetS criterion BMI 25–35 kg/m^2^	8 weeks	Mediterranean Diet (fibre: 19.3 g/1000 kcal) *vs* Control Diet (fibre: 8.1 g/ 1000 kcal)	Mediterranean diet compared to control diet ↑*Intestinimonas butyriciproducens* ↑*Akkermansia muciniphila* ↑Plasma butyric acid ↓Postprandial glucose ↓Postprandial insulin ↑OGIS
[23]	Parallel	43 individuals with T2D BMI 25–35 kg/m^2^	12 weeks	High-fibre Diet (fibre:37.1 g) *vs* Control Diet (fibre:16.1 g)	High-fibre diet compared to control diet ↑Faecal butyrate ↓HbA1c ↓Fasting glucose
[24]	Parallel	20 individuals with MetS BMI 30-40Kg/m^2^	1 year	Mediterranean Diet (fibre: 12.9 ± 0.2 g/Kcal, mainly from vegetables) *vs* High-fibre Diet (fibre: 14.1 ± 0.2 g/1000 kcal, mainly form wholegrains)	Mediterranean diet ↑*Roseburia* ↓*Prevotella* ↑ISI High-fibre diet ↓*Roseburia* ↑*Prevotella* ↑ISI
[25]	Crossover	19 individuals with MetS BMI 25,9–41 kg/m^2^	4 weeks	Diet enriched with Arabinoxylan and Resistant starch (fibre:64 g) *vs* Western diet (fibre:17.6 g)	Healthy-carbohydrate diet enriched with arabinoxylan and resistant starch compared to Western diet style ↑*Bifidobacteria* ↑Faecal SCFA ↑Faecal Butyrate
[26]	Parallel	40 individuals with MetS BMI 25–35 kg/m^2^	12 weeks	Wholegrain diet (total fibre: 40 g; fibre from cereal:28.9 g) *vs* Refined cereal diet (total fibre: 22.1 g; fibre from cereal 11.8 g)	Wholegrain diet compared to refined cereal diet ↑Plasma propionate ↓Postprandial insulin
[27]	Crossover	39 healthy individuals BMI 18–28 kg/m^2^	3 days	Barley kernel-based bread (fibre:37.6 g) *vs* White wheat bread (fibre:9.1 g)	Barley kernel-based bread compared to white wheat bread ↑*Prevotella:Bacteroides* ↓Postprandial glucose
[28]	Crossover	39 healthy individuals BMI 18–28 kg/m^2^	3 days	Barley kernel-based bread (fibre:37,6 g) *vs* White wheat bread (fibre:9.1 g)	Barley kernel-based bread compared to white wheat bread ↑Plasma SCFA ↓Glucose ↓Insulin

*BMI* Body mass index; *HbA1c* Glycated hemoglobin; *ISI* Insulin sensitivity index; *MetS* Metabolic syndrome; *OGIS* Oral glucose insulin sensitivity; *SCFA* Short-chain fatty acids.

As for high-fibre diets, a short-term randomized controlled clinical trials (4 days) in a small group of healthy individuals compared the prebiotic effect of a Western diet (WD) –based on the consumption of animal foods (meat, eggs and cheese)—with low fibre intake (9.36 ± 2.1 g/1000 kcal) or a plant-based diet, rich in fibre (25.6 ± 1.1 g/1000 kcal) from wholegrains, legumes, fruits and vegetables. The results showed that plant-based diet increased plant-polysaccharide metabolizing bacteria (*Prevotella* and *Roseburia)* with an higher concentration of faecal SCFA in particular butyrate, as compared to Western diet [21].

More recently, an 8-week Mediterranean diet, rich in fibre (fibre: 19.3 ± 3.1 g/1000 kcal), has shown to increase *Intestinimonas butyriciproducens* and *Akkermansia muciniphila* abundance, and postprandial plasma butyrate concentrations, with an improvement in postprandial glucose and insulin sensitivity in individuals with high cardiometabolic risk, compared to Control Diet (fibre: 8.1 ± 2.3 g/1000 kcal). Interestingly, butyrate concentrations directly correlated with postprandial insulin sensitivity, evaluated by OGIS [22].

Even in individuals with T2D, a 12-week high-fibre diet (fibre: 37.1 ± 1.9 g) has shown to increase faecal butyrate concentrations that associated with the reduction in fasting glucose and HbA1c concentrations [23].

In addition, another trial in a small group of individuals with metabolic syndrome has demonstrated that plant-based diets may selectively increase some bacterial species, depending on the type of fibres consumed. Indeed, in a long-term clinic trial (1 year), a high-fibre diet (fibre: 14.1 ± 0.2 g/1000 kcal mainly from wholegrains) increased *Prevotella*, while Mediterranean Diet (fibre: 12.9 ± 0.2 g/1000 kcal, mainly from vegetables and nuts) enhanced *Roseburia* abundance. Meanwhile both diets increased *Faecalibacterium prausnitzii*. Interestingly, an improved insulin sensitivity was observed after both diets [24].

Among high-fibre foods, wholegrains have been extensively studied by virtue of their high fibre content. Indeed, it has been shown that cereal fibre (arabinoxylans and bran) is highly fermentable and may increase faecal short-chain fatty acid concentrations after just 4 weeks [25].

Moreover, a 12-week wholegrain-based diet (cereal fibre: 28.9 ± 1.1 g/day) has shown to increase plasma propionate concentration compared to a refined-cereal-based diet used as control (cereal fibre: 11.8 ± 0.4 g/day), and this increase correlated with an improved insulin postprandial response in individuals with Metabolic Syndrome [26].

As for the effect of specific foods, two studies [27, 28] have showed that the consumption of barley kernel-based bread (fibre: 37.6 g/day) increased *Prevotella* while reduced *Bacteroides* after only 3 days compared to wheat bread (fibre: 9.1 g/day) in healthy volunteers. This change was associated with the reduction in postprandial glucose response [27, 28] which correlated with the increased total serum SCFA concentration [28].

In conclusion, although many studies have been performed in small groups and with a short duration, the results from the main intervention trials (Table 2) indicate that high-fibre diets and fibre-rich foods are able to improve glucose metabolism and this improvement is associated with changes in gut microbiota and increased SCFA concentration.

## Possible mechanisms of action

Dietary fibre has shown to influence glucose metabolism by several mechanisms in healthy individuals and people with T2D, mainly driven by its functional properties (viscosity, water solubility, and fermentation rate) [29]. New insights into the capacity of dietary fibre to modulate microbial composition and activity triggered more attention on microbial metabolites, particularly SCFA.

**Fig. 2:**
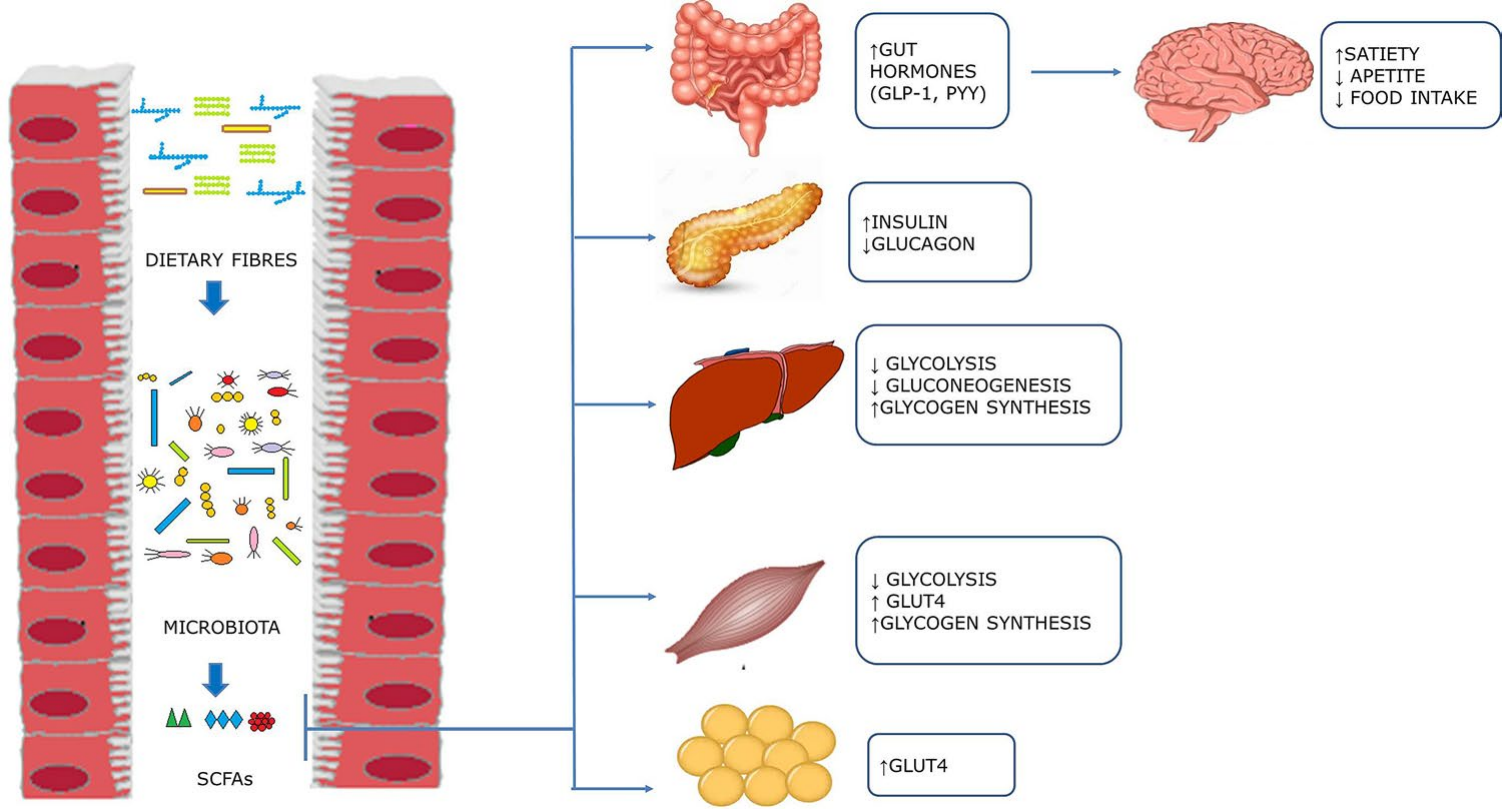
Main mechanisms of action of short-chain fatty acids on glucose metabolism. *GLP-1* Glucagon-like peptide‑1, *GLUT-4* Activated glucose transporter protein-4, *PYY* Peptide YY, *SCFAs* Short-chain fatty acids

Accumulating evidence supports local—meaning in gastrointestinal tract—and systemic effect of SCFA that might affect glucose metabolism (Fig. 2).

Studies in vitro and in vivo showed that SCFA are potent secretagogues for glucagon-like peptide‑1 (GLP-1) and peptide YY (PYY) that increase satiety feeling through the gut–brain axis. As a consequence, they might indirectly reduce appetite and consequent food intake, thus preventing body weight gain, a well-known risk factor T2D. Moreover, SCFA might regulate blood glucose concentrations through a GLP-1-mediated increase in insulin secretion [30].

In the liver, SCFA has shown to decrease glycolysis and gluconeogenesis, and to increase glycogen synthesis and fatty acids oxidation [30–34].

As for extra intestinal effects, SCFA have shown to improve glucose uptake in skeletal muscle and adipose tissue by increasing the expression of GLUT4, through AMP Kinase (AMPK) activity. In addition, in the skeletal muscle, SCFA reduce glycolysis with a consequent accumulation of glucose-6-phosphate and increase in glycogen synthesis [31–37].

The possible effects of SCFA, in particular butyrate and propionate, in the modulation of glucose metabolism in humans, pushed to carry out intervention studies with propionate and butyrate supplementation. However, the evidence is not conclusive since the studies are few and have been performed in small groups of individuals. Nevertheless, they seem to indicate that: 1) an inulin-propionate supplementation (10 g/day) increases GLP-1 and PYY, and reduces food intake [38, 39] thus contributing to body weight regulation, and 2) sodium butyrate supplementation (4 g/day) improves insulin sensitivity only in lean subjects and not in individuals with metabolic syndrome [40].

## Conclusion

Based on the current evidence, gut microbiota might play a pivotal role in the regulation of glucose metabolism and, therefore, may be associated with a reduction in type 2 diabetes risk. As already mentioned in Introduction, this association may be mediated by several mechanisms (i.e. maintenance of the integrity of intestinal barrier, reduced endotoxemia, and production of microbial metabolites). Some bacteria (i.e. *Akkermansia muciniphila, Lactobacillus* and *Bifidobacteria*) may reduce intestinal permeability and inflammation [5, 41]. As for microbial metabolites, short-chain fatty acids (SCFAs) have shown pleiotropic effects in different sites that regulate glucose metabolism. SCFAs acids are produced by microbiota through the fermentation of dietary fibre. At the same time, a fibre-rich diet has shown a prebiotic effect towards SCFA-producing microbial species (i.e. *Roseburia, Faecalibacterium prausnitzii, Prevotella*) [42, 43]. This evidence supports microbiota as key actor in the interplay between fibre intake and the prevention and management of metabolic diseases.

Therefore, the well-known effect of dietary fibres in preventing T2D [44] may be explained, at least in part, also throughout this mechanism. Unfortunately, no studies have evaluated specifically whether soluble and insoluble fibres may differently affect microbiota. According to fibre used in the studies (i.e. β-glucan and arabinoxylans from wholegrains, pectins from fruit, vegetables, and legumes, and resistant starch), it could be hypothesized that soluble readily fermented fibres might be more effective in mediating the interplay between diet and microbiota in the improvement of glucose homeostasis than other types of fibres [29]. Conversely, insoluble fibre might reduce T2D risk through other mechanisms (i.e. promoting body weight management, increasing faecal glucose-excretion) [29, 45].

Therefore, individuals with prediabetes or diabetes should be advised to increase dietary fibre intake, favouring the consumption of wholegrain, legumes, fruit and vegetables. So far, no recommendation can be stated for fibre supplements or SCFA-based formulations.

Another point to consider is that the relationship between fibre intake, SCFAs, and microbiota has been observed in the context of traditional diets. More extreme diets (i.e. carbohydrate-restricted and ketogenic diets) have shown dramatic effects on microbiota composition and activity. Indeed, a reduction in SCFA-producing microbial species and *Bifidobacteria*, and SCFAs concentrations has been observed after short-term carbohydrate-restricted diets [46, 47]. The long-term effects of these diets in the relationship between microbiota and health status need further investigations.

In conclusion, increasing daily fibre intake in the context of a healthy dietary pattern might be a valid tool to improve microbiota composition and activity to prevent metabolic diseases.
